# Editorial: Vestibular migraine: pathophysiology, diagnosis, and management

**DOI:** 10.3389/fneur.2026.1891661

**Published:** 2026-06-10

**Authors:** Alexander A. Tarnutzer, Adina Maria Roceanu, Madalina Georgescu, John Waterston, Michael von Brevern, Thomas Lempert

**Affiliations:** 1 Neurology, Cantonal Hospital of Baden, Baden, Switzerland; 2 Faculty of Medicine, University of Zurich, Zurich, Switzerland; 3 University Emergency Hospital Bucharest, Bucharest, Romania; 4 Carol Davila University of Medicine and Pharmacy, Bucharest, Romania; 5 Department of Neuroscience, Central Clinical School, Monash University, Melbourne, VIC, Australia; 6 Department of Neurology, Charité, Berlin, Germany; 7 Department of Neurology, Alexianer St. Gertrauden -Krankenhaus, Berlin, Germany

**Keywords:** differential diagnosis, headache, management, pathophysiology, vertigo, vestibular migraine (VM)

Vestibular migraine is an increasingly recognized cause of episodic vertigo and dizziness, currently considered as one of the most common vestibular disorders. Fueled by established and widely accepted diagnostic criteria promoted by both the International Headache Society and the Bárány Society (1), a growing number of basic and translational research studies and clinical trials have advanced our understanding of this entity. Nonetheless, significant challenges remain, including the very heterogeneous spectrum of clinical presentations, the lack of biomarkers, the overlap to other, less common vestibular disorders and evolving entities such as “chronic vestibular migraine” (2). With the underlying pathophysiology of vestibular migraine still being unknown, targeted treatment strategies are limited and current treatment is not yet evidence based. Thus, there is an urgent need for further research in the field to improve diagnosis and management.

In this topic collection of research articles, both new diagnostic approaches, pathophysiological links to other disorders, clinical features and potential biomarkers are presented, further advancing our understanding of vestibular migraine.

Improving diagnostic accuracy in patients presenting with episodic vestibular syndromes is an urgent need. One article of this topical Research Topic proposed a diagnostic prediction model for vestibular migraine to facilitate the diagnostic process (Zhang et al.). With the help of multimodal data, including clinical features, vestibular function tests, hematological indicators, contrast transthoracic echocardiography, and psychological assessments, and the use of logistic regression a prediction model was generated and evaluated. Eventually, the final model (including six variables: body-mass index (BMI), presence of emotional triggers, insomnia triggers, history of motion sickness, and abnormal otoacoustic emissions) achieved high accuracy. While preliminary, multimodal prediction models as the one presented here are promising clinical tools to improve VM diagnostic accuracy, but await validation for generalizability in larger, prospective trials.

The complex relationship between Menière's disease (MD) and vestibular migraine (VM) is reviewed in another study included in this Research Topic. Whether MD and VM are overlapping disorders (sharing some fundamental multifactorial processes) or completely separate entities is still debated. In the work of Sun et al. a comparative analysis of MD and VM across multiple dimensions, including pathogenesis, clinical manifestations, and diagnostic criteria is provided. The diagnostic criteria (and their evolution), differences in presenting findings and in successful treatment strategies are accurately set out. Although MD and VM may demonstrate clinically overlapping symptoms and/or at least partially shared imaging findings, the genetic and molecular mechanisms are pointing to two different entities, as emphasized by the authors. Prioritizing research on potential imaging biomarkers, serum biomarkers, suitable animal models and machine learning for integrating multimodal input may be a promising strategy to improve in the distinction between VM and MD.

A special focus to develop simple bedside biomarkers to improve early diagnosis of vestibular migraine is put in another study in this Research Topic. There have been reports of abnormal visuo-vestibular interaction in the setting of vestibular migraine. Cortese et al. examined the cervical vestibular evoked myogenic potentials before and after unidirectional visual motion stimuli delivered via virtual reality goggles in subjects with vestibular migraine. They found significant degrees of interaural asymmetry in the VM group, which decreased following prolonged visual stimulation. However, there was no significant change in amplitude, limiting the use of this test as a biomarker.

Various disorders causing dizziness are associated with migraine including Menière's disease, BPPV and persistent postural-perceptual dizziness (PPPD). The association of postural tachycardia syndrome (POTS) with migraine is less well-known and was analyzed in depth by Sekhon et al. in this series. In a cohort of 80 POTS patients, they identified 84% with migraine and 30% with vestibular migraine. The strength of the association may reflect some selective referral to a specialized center. Nevertheless, the clinical takeaway is that doctors caring for patients with either of the two conditions have to be aware of this comorbidity to guide treatment appropriately.

Likewise, the processing of visual input may be significantly altered in VM patients, biasing verticality perception. Such abnormalities can be detected using either tests of static subjective visual vertical (SSVV) or dynamic SVV (DSVV) or the Rod-and-Frame test (RFT). Both the DSVV and the RFT probe visuo-spatial integration by increasing visual dependence. Whereas, for the DSVV a moving background creates an optokinetic effect, the patient is immersed in a tilted rectangular visual box for the RFT. Using a new virtual reality (VR)—based protocol that combined all these test (SVV, DSVV, RFT)—allowing for their simultaneous use—Korkmaz et al. characterized the visuo-vestibular integration in both VM patients, migraine patients without vestibular symptoms and healthy controls. These VR-based tests revealed that VM resulted in higher visual dependence and more severely impaired multisensory integration. This was especially true for dynamic and visually complex situations. Combining static, dynamic, and contextual assessments improved the diagnostic sensitivity in this cohort. These findings have implications on treatment strategies, supporting personalized vestibular rehabilitation approaches to reduce visual dependence and to enhance sensory balance in VM patients.

Vestibular migraine may also negatively impact adaptive learning due to increased perceptual uncertainty related to the associated dizziness, as proposed by Sharif et al. To assess this hypothesis, patients with either acute or chronic vestibular migraine presentations completed the IOWA gambling task. In general, vestibular migraine patients demonstrated higher-risk taking behavior than healthy controls. In those patients with acute vestibular migraine, a significant correlation between impaired learning and the level of dizziness was detected. These findings provide important insights into how vestibular migraine can affect behavioral adaption in patients, either directly through altered perception or indirectly by impacting cognitive processes that can result in maladaptive behavior (Sharif et al.).

An area mostly neglected in vestibular migraine research are the presumably symptom-free “interictal” intervals. While ictal vestibular migraine symptoms have caught more attention and are considered to be more valuable for making the diagnosis right, clinically relevant interictal complaints may be overlooked. With the aim to promote more awareness of interictal symptoms, Rust and Palla propose a spectrum-based view of vestibular migraine, in which episodes of symptom exacerbation and persistent interictal symptoms represent different clinical expressions within the same disorder. In this framework, persistent sensory hypersensitivity and non-specific dizziness are considered the background noise of the disorder. This prompted the authors to promote diagnostic criteria for “chronic vestibular migraine” based on symptom frequency. From a practical viewpoint, prospective validation of such preliminary criteria will be needed to assess their value in the clinical setting.

Likewise, the natural history of vestibular migraine has received little attention. A multicenter cross-sectional study by Celebisoy et al. examined the prognosis and the natural course of vestibular migraine. Median attack frequency and severity of vertigo had significantly dropped after a follow-up of at least 4 years, although patients were not on preventive treatment at time of reevaluation. Patients with aural symptoms and women in menopause had a higher risk of no improvement of vertigo frequency and severity (Celebisoy et al.). Many open label studies and retrospective case series describe improvement of vestibular migraine with various preventive drugs. Possibly, most of these studies just describe a placebo effect and the natural course. Thus, knowledge about the evolution of symptoms of vestibular migraine underlines the need for well-conducted randomized controlled trails to assess efficacy of treatment.

In summary, recent evidence published in this Research Topic further underlines the importance of expanding our view and concepts of vestibular migraine, both with regards to clinical presentations, biomarkers, overlap with other disorders and its impact on other systems. At the same time, also gaps in knowledge are identified and future research strategies are outlined, further advancing our understanding of VM. This includes increased awareness of interictal signs and symptoms and the distinction between the disorder's natural history and response to treatment, remaining cautious and avoiding overestimating the impact of preventive drug treatment.
